# Differential diagnosis of posterior scalp hair loss

**DOI:** 10.1007/s00403-024-03485-0

**Published:** 2024-11-02

**Authors:** Shaveonté Graham, Jorge Larrondo, Ivie Obeime, Amy McMichael

**Affiliations:** 1 Wright State University Boonshoft School of Medicine, Dayton, OH USA; 2 Department of Dermatology, Wake Forest University School of Medicine, Winston-Salem, NC USA

**Keywords:** Traction alopecia, Alopecia areata, Hair breakage, Lichen planopilaris, Frontal fibrosing alopecia, Acne keloidalis nuchae, Occipital scalp, Central centrifugal cicatricial alopecia, CCCA, Dissecting cellulitis, And folliculitis decalvans, Posterior scalp

## Introduction

Hair loss is a major stressor that affects people of all ages. It can affect any area of the scalp, but little attention has been given to disorders primarily affecting the occipital scalp. The etiology of hair loss affecting primarily the occipital scalp is unknown, however, we can postulate a few explanations, including that the occiput is an area of a lot of movement, which can irritate the hair follicles, there is often occlusion of the area with clothing, and finally, there may be a predilection of hair follicles in this area that are at risk in the same way the hair follicles on the vertex and frontal scalp are at risk for pattern hair loss in a way that we don’t yet understand. The occipital scalp is a difficult area to evaluate as lesions frequently go unnoticed as they are often camouflaged by the remaining hair on the scalp. If a patient desires to wear a style that might accidentally reveal the posterior scalp loss, anxiety and embarrassment can ensue, in addition to a negative effect on quality of life [1]. There are limited data on the conditions that favor the posterior scalp, potentially leading to missed diagnoses, inappropriate treatment, and poor outcomes.

The goal of this review is to describe forms of hair loss that often occur solely on the occiput, providing a differential for the area, so these diagnoses would not be missed early on, which often happens. The clinical exam, trichoscopic, and biopsy findings, as well as the diagnostic features that distinguish these posterior scalp conditions will be reviewed. The forms of occipital alopecia discussed include: traction alopecia (TA), alopecia areata (AA), hair breakage, lichen planopilaris (LPP), frontal fibrosing alopecia (FFA), central centrifugal cicatricial alopecia (CCCA), acne keloidalis nuchae (AKN), dissecting cellulitis (DC), and folliculitis decalvans (FD).

## Methods

A literature search utilizing PubMed was conducted. A keyword search for traction alopecia, hair breakage, hair fragility, alopecia areata, acne keloidalis nuchae, frontal fibrosing alopecia, lichen planopilaris, central centrifugal cicatricial alopecia, dissecting cellulitis, folliculitis decalvans, and occipital scalp alopecia resulted a total of 16,479 articles after limiting results to English. Articles that were the incorrect topic, did not include occipital scalp, and were treatment focused were excluded, resulting in 46 articles. Results included case reports and series, reviews, and meta-analyses.

## Results

### Nonscarring conditions

#### Traction alopecia (TA)

**Fig. 1:**
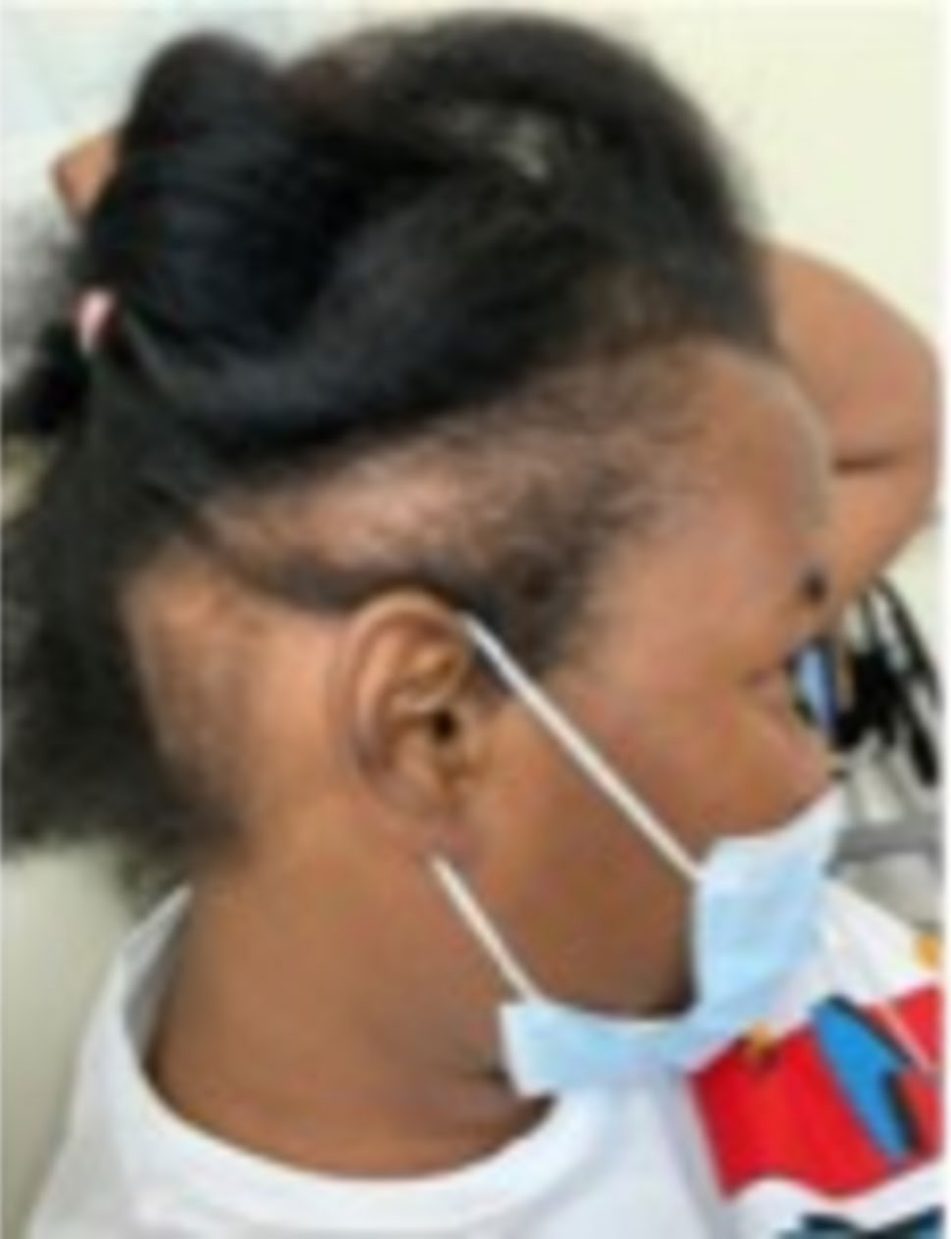
Occipital traction alopecia

**Fig. 2:**
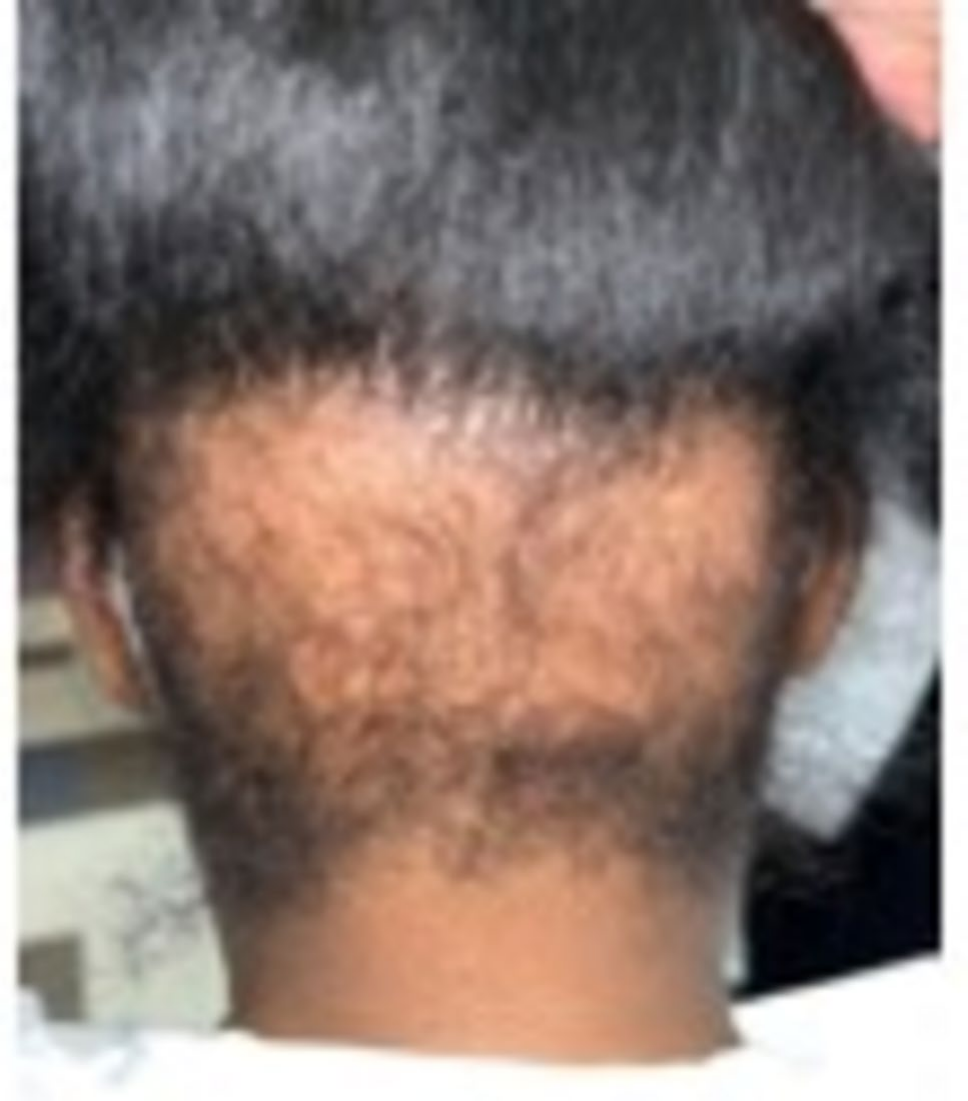
Occipital traction alopecia

**Fig. 3:**
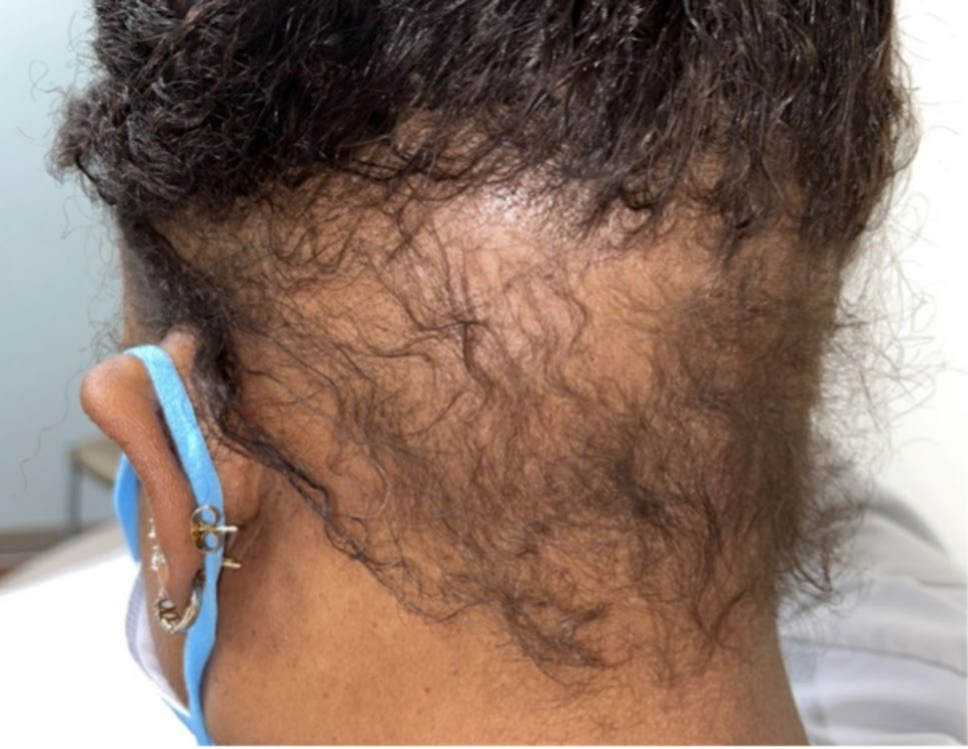
Biopsy proven occipital traction alopecia

TA, a nonscarring form of hair loss, commonly affects women of African descent who wear traumatic hairstyles for prolonged periods of time. Khumalo reported the prevalence of TA to be 17.1% in girls, with occurrence increasing with age and in those with chemically relaxed hair [2]. The prevalence in women was 31.7% versus 2.3% in men [2]. The frontal hairline is the most commonly affected area, however, TA can occur in the inferior occipital scalp as the only affected location [3–5]. TA is classified as marginal (most common and affecting the frontal and temporal scalp) (Fig. 1) and nonmarginal (including the occipital scalp) (Figs. 2, 3). Either form of TA can be caused by hairstyles such as tight buns, ponytails, weaves, hair extensions, tight braids and locks [6–8]. Traction alopecia on the occipital scalp caused by tight buns is known as chignon alopecia. All forms of TA result from excessive pulling forces and duration of tight hairstyles, which lead to mechanical damage of the hair follicles and induces an inflammatory response and ultimate hair loss [2, 9, 10].

##### Clinical exam

Acutely, patients may present with tenderness, itching, paresthesias, and headache. In early stages, perifollicular erythema can develop into folliculitis characterized by perifollicular pustules and papules [2]. Chronic changes include patches of non-scarring hair loss along the area undergoing tension, with broken hairs and/or follicular pustules [4]. The fringe sign, observed on the frontal or posterior scalp, is the presence of short intermediate or terminal hairs bordering areas of hair loss [3, 8] and can be helpful in differentiating TA from other types of hair loss such as FFA or AA ophiasis type [7]. Long-term use of traumatic hairstyles may present with irreversible scarring alopecia, little to no inflammation, and decreased follicular markings. [3]

##### Trichoscopy and pathology

**Fig. 4:**
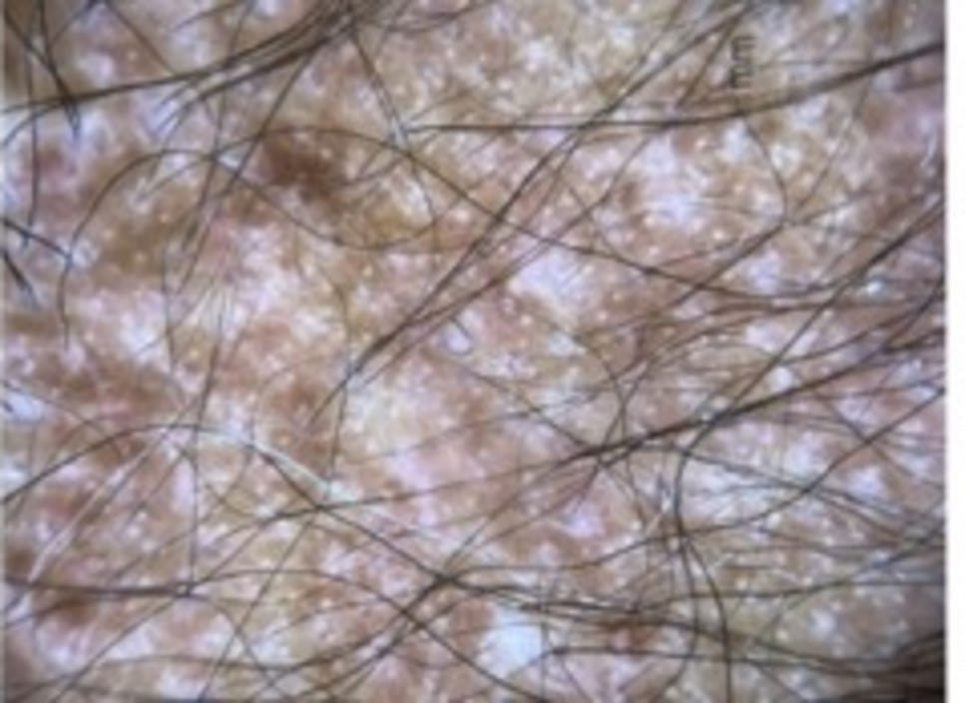
Perifollicular erythema and hair casts. Reduction in hair density, hair diameter, diversity, empty follicles, and vellus hairs in occipital traction alopecia

**Fig. 5:**
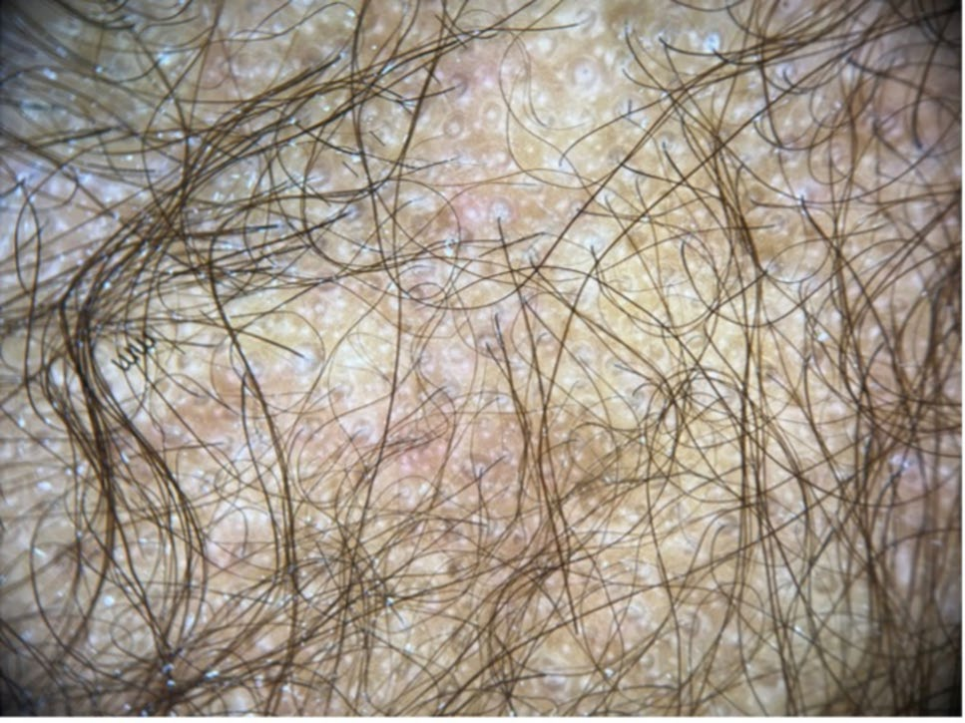
Perifollicular erythema, loss of follicular openings, broken hairs, and yellow dots in traction alopecia

**Fig. 6:**
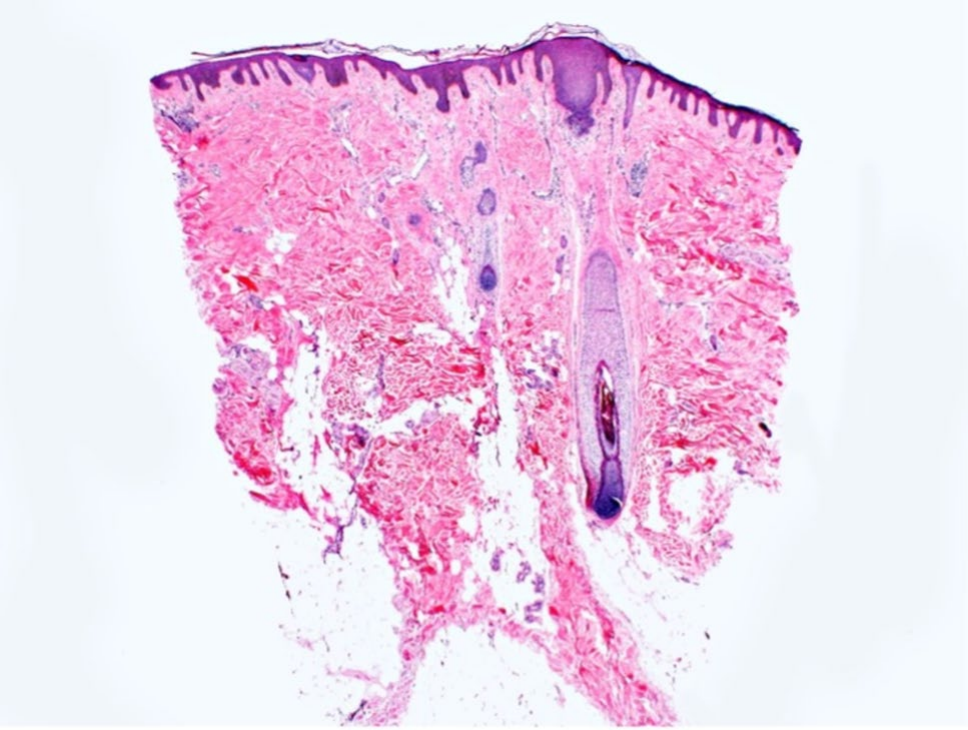
Reduction of terminal hairs replaced by connective tissue, and a reduction of sebaceous glands in traction alopecia

Trichoscopy can help diagnose TA, however, findings can vary by disease duration. Acute TA shows perifollicular erythema and hair casts [11]. Chronic TA includes loss of follicular openings, broken hairs and yellow dots [11]. Both forms have reduction in hair density, hair diameter diversity, empty follicles, and vellus hairs [11] (Figs. 4, 5). If occipital TA is suspected, but not clear clinically or trichoscopically, a biopsy may be required. On pathology, early TA resembles trichotillomania characterized by an increased number of telogen and catagen hair follicles, a normal number of hair follicles, pigment casts, intact sebaceous glands, and trichomalacia [3, 12]. Later stages show reduction of terminal hairs that are replaced by connective tissue, vellus hairs, and reduction of sebaceous glands [3, 12] (Fig. 6).

##### Differential diagnosis

Differentials for acute TA with occipital compromise include trichotillomania, pressure alopecia, and AA. Hair casts are absent in trichotillomania [13]. For chronic TA, diffuse, incognito, and ophiasis AA including and occipital FFA should be considered. Follicular markings or ostia are the most distinguishing factor. While decreased in chronic TA, they are retained in ophiasis AA and absent is FFA [3]. Other distinguishing clues include scalp color, eyebrows, perifollicular erythema and scale, and the fringe sign (Table 1). TA has normal scalp color, retained eyebrows, perifollicular erythema, scale, and fringe sign; AA ophiasis type may have peach-colored scalp, possible eyebrows involvement, absent perifollicular erythema and scale, and could mimic fringe sign, although not symmetric or bilateral; lastly FFA has pale and atrophic scalp with accentuation of veins, possible eyebrow involvement, present perifollicular erythema, slight or absent scale, and absent fringe sign [3].

**Table 1:** Distinguishing factors of occipital scalp conditions

Condition	Distinguishing factors
Traction alopecia	Presence of the “fringe sing” Reduction of follicular markings Perifollicular erythema and scale –early TA Intact sebaceous glands
Alopecia areata	Presence of exclamation point hairs, broken hairs, black dots, and tapering hair Presence of follicular markings No perifollicular erythema and scale Peribulbar infiltrate Nail pitting
Hair breakage and fragility	Uneven, broken hairs Dry, brittle hair Trichorhexxis nodosa on hair shaft microscopy Broom-stick like ends of hair fragments
Lichen planopilaris	Multiple patches in the scalp Absence of vellus hairs Peripilar casts Absence of sebaceous glands Perifollicular lichenoid infiltrate with fibrosis More severe inflammatory infiltrate, less apoptosis
Frontal fibrosing alopecia	Eyebrow, facial, and body hair involvement Presence of the lonely hair sign Absence of vellus hair Presence of the pseudo-fringe sign Absence of follicular markings Minimal inflammatory infiltrate, more apoptosis Loss of sebaceous glands –early-stage
Central centrifugal cicatricial alopecia	Vertex involvement Follicular drop out Peripilar white–gray halo Perifollicular hyperkeratosis Positive family history Premature desquamation of the inner root sheath
Acne keloidalis nuchae	Absence of ingrown hairs Follicular papules and nodules
Dissecting Cellulitis	Stage 1–follicular and perifollicular lymphocytic infiltrates on the lower parts of terminal follicles, yellow dots, broken hairs, black dots, and exclamation mark hairs Stage 2–three-dimensional yellow dots with “soap bubble” -like appearance, yellow structureless areas known as “lakes of pus” and pinpoint-like vessels with a whitish halo Stage 3–white areas lacking follicular openings that represent tissue fibrosis, cutaneous clefts with emerging hairs may emerge
Folliculitis Decalvans	Clinical hallmark–development of scarred areas and areas of follicular pustules Tricoscopic hallmark–presence of multiple hairs, generally 5–20, emerging from one single dilated follicular orifice

#### Alopecia areata

AA is an autoimmune form of nonscarring hair loss that affects any area of the scalp. When the occipital scalp is affected, the designation ophiasis pattern is used. Ophiasis pattern classically presents as a band-like hair loss along the hairline of the posterior auricular and occipital scalp [14]. In a retrospective chart review [15], it was determined that 15.1% of 198 patients presented with ophiasis pattern AA [15]. This form is particularly difficult to treat, so early recognition and treatment is imperative.

##### Clinical exam

**Fig. 7:**
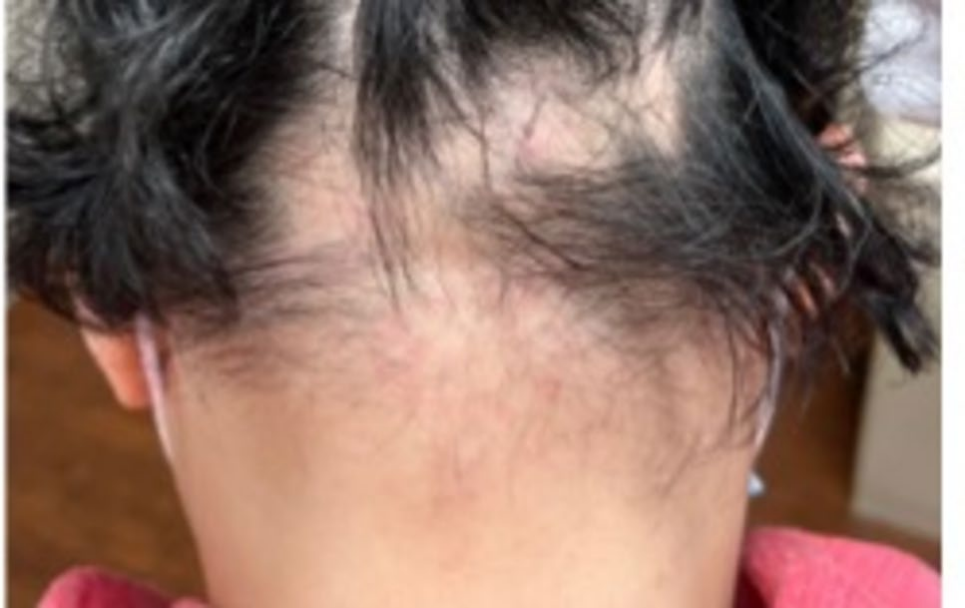
Occipital alopecia areata

**Fig. 8:**
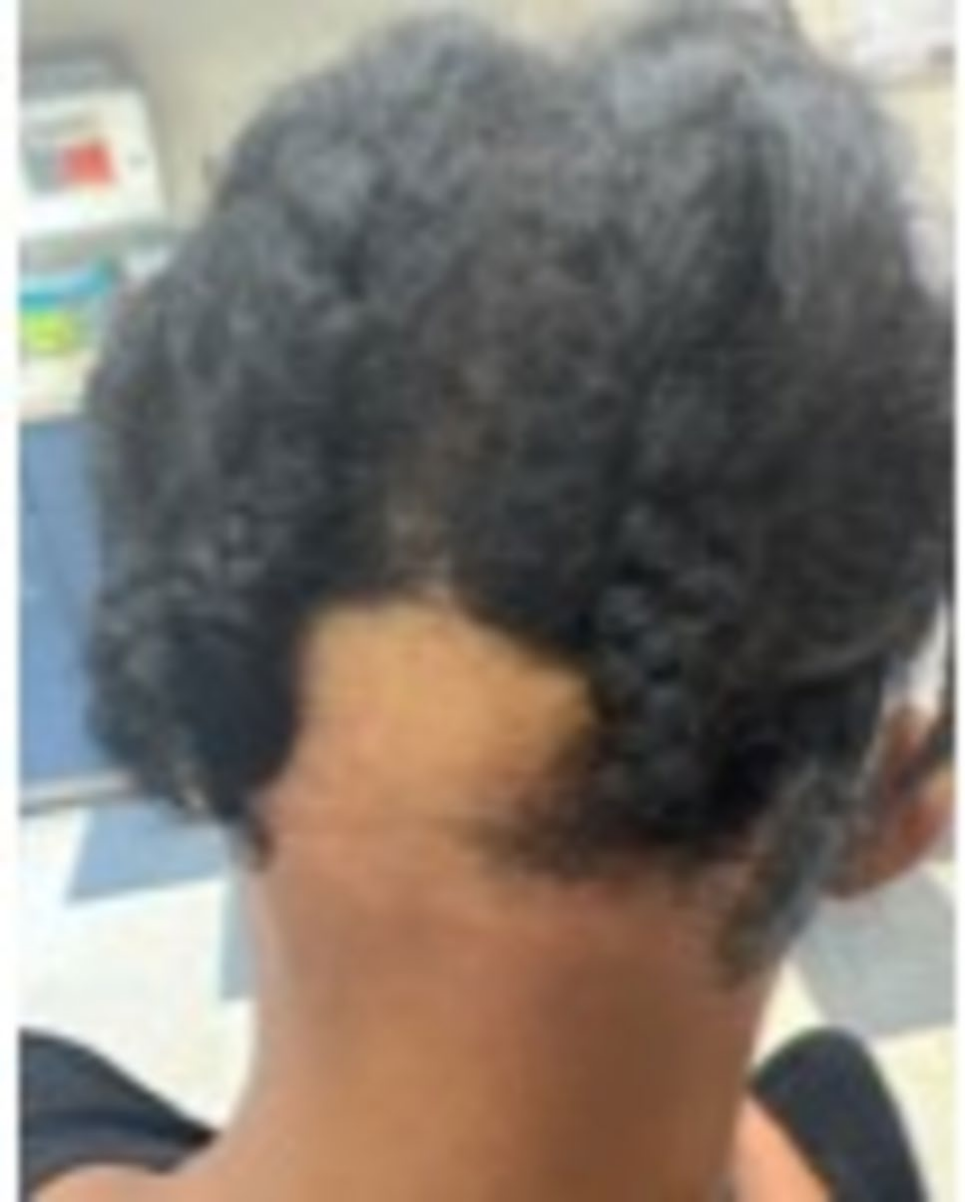
Occipital alopecia areata

**Fig. 9:**
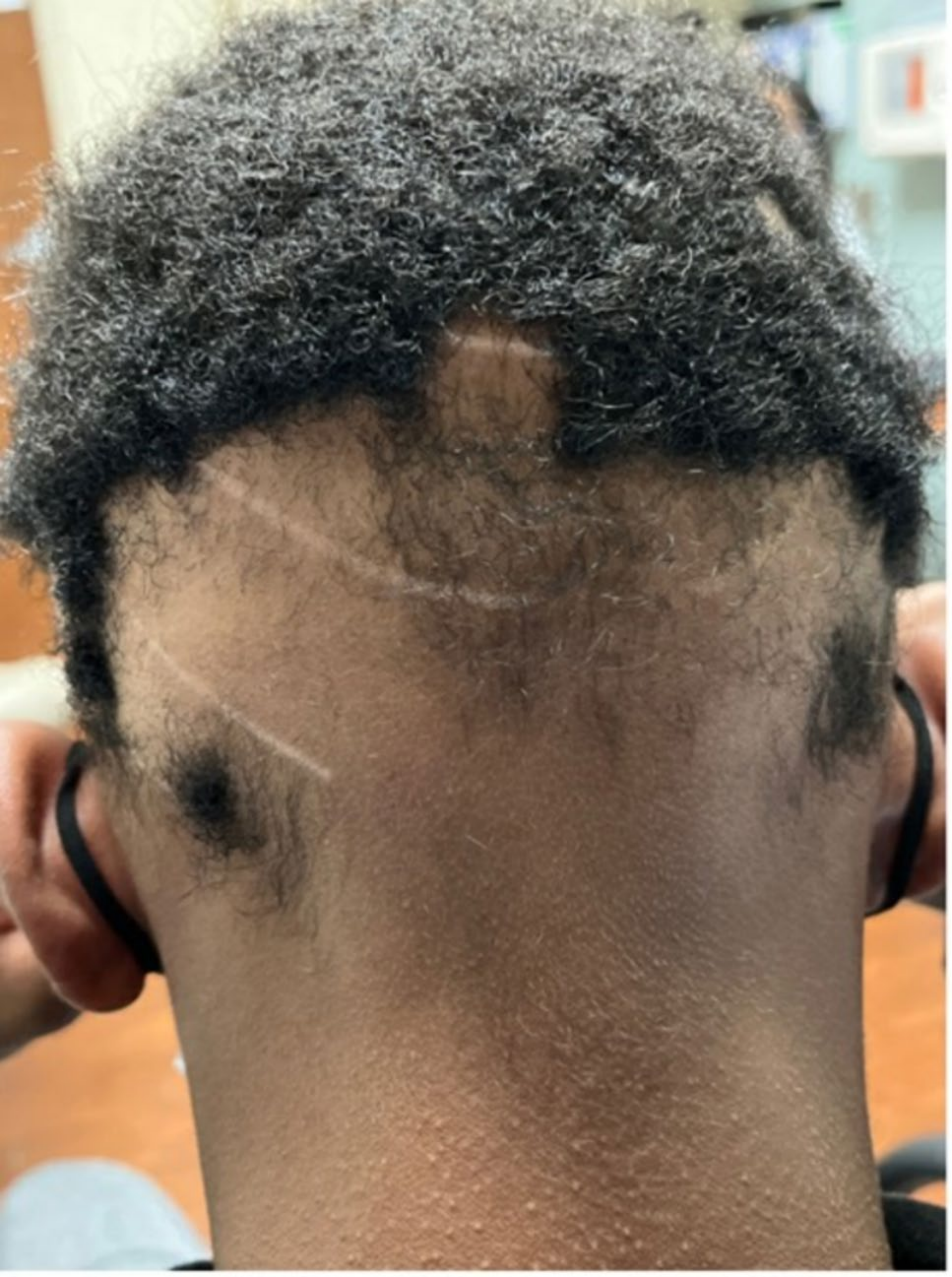
Occipital alopecia areata

Acute AA presents with well-defined alopecic patches and a positive pull test, a marker of a dystrophic anagen phase [14] (Figs. 7, 8, 9). The underlying affected skin typically appears normal without signs of inflammation or scarring, though there may be mild erythema [8]. AA is usually asymptomatic, but a small percentage of patients may present with pruritus or tingling [14]. Patients can also present with nail dystrophy consisting of pitting, and trachyonychia [16].

##### Trichoscopy and pathology

**Fig. 10:**
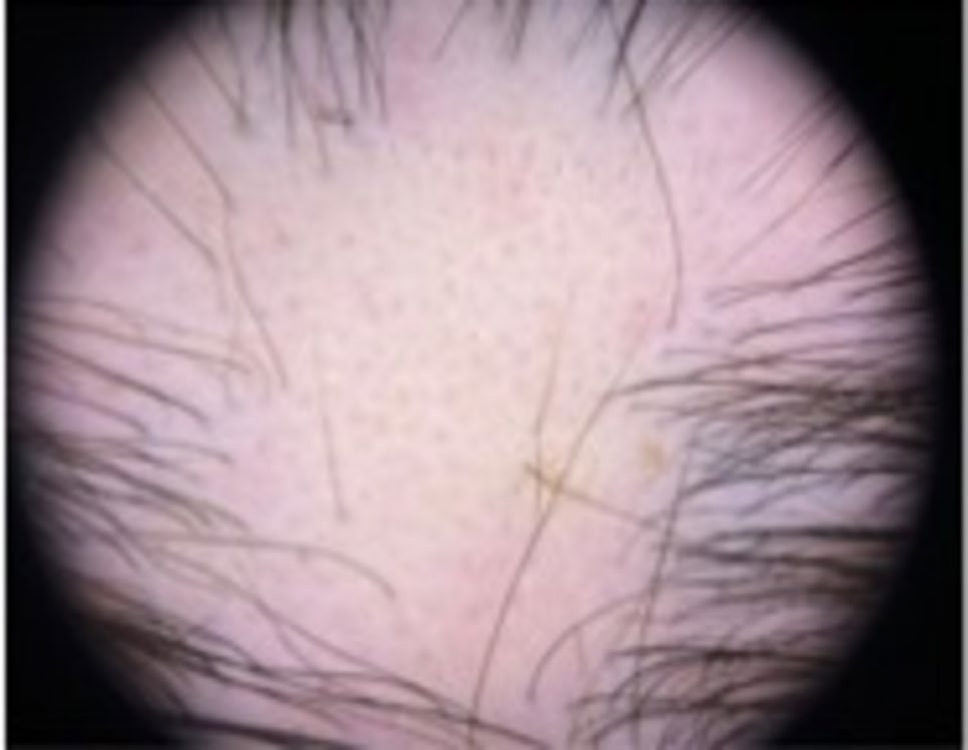
Exclamation mark hairs, dystrophic hairs, black dots, broken hairs, and short vellus hairs in alopecia areata

Trichoscopy findings include exclamation mark hairs, dystrophic hairs, black dots, circle hairs, yellow dots, broken hairs, short vellus hairs, upright regrowing hairs, pigtail hairs, and tapered hairs [14, 17] (Fig. 10). Exclamation mark hairs, found during active phases, is characterized by a narrowed hair root, suggesting defective anchoring of the hair within the follicle [14]. On the rare occasion that pathology is required to distinguish AA from another form of occipital hair loss, the classic appearance is a peribulbar infiltrate likened to a “swarm of bees” with a lymphocytic infiltrate around and within the hair bulb [14, 18]. There may be increased follicles in catagen or early telogen with miniaturization [14, 18]. The chronic stage demonstrates follicles in telogen phase or miniaturized with anagen morphology, resulting in appearance of non-inflammatory alopecia with preservation of the sebaceous gland [18]. Pigmentary incontinence in follicular dermal papillae and degeneration of the hair bulb epithelium may also be present [18].

##### Differential diagnosis

To differentiate AA from other nonscarring alopecias, miniaturized hairs and increased catagen/telogen with mild to no inflammation can be seen in androgenetic alopecia and chronic TA.

#### Hair breakage and fragility

Hair breakage and fragility can occur due to genetic predisposition in association with weathering from various haircare practices [19, 20] or trauma to the scalp. There were no measurable cysteine changes found in Afro-textured hair, suggesting that breakage is due to structural damage, such as breaks, partial breaks, knots, and longitudinal splits [21], which often occur with normal grooming. Trichorrhexis nodosa is a common hair shaft disorder that stems from damage or loss of the cuticular layer leading to fragility and longitudinal fraying of the hair shaft [22]. This is seen in traumatic haircare practices such as chemical straightening and dye treatments, aggressive brushing or combing [22, 23], or conditions such as seborrheic dermatitis [23], tinea capitis (TC) [23], and early CCCA) [25]. Heat treatments like blow drying and straightening tools used over time may cause hair shaft damage leading to hair breakage [23]. Lastly, monilethrix is a rare hair shaft disorder characterized by hair breakage before reaching more than a few inches in length.

##### Clinical exam

**Fig. 11:**
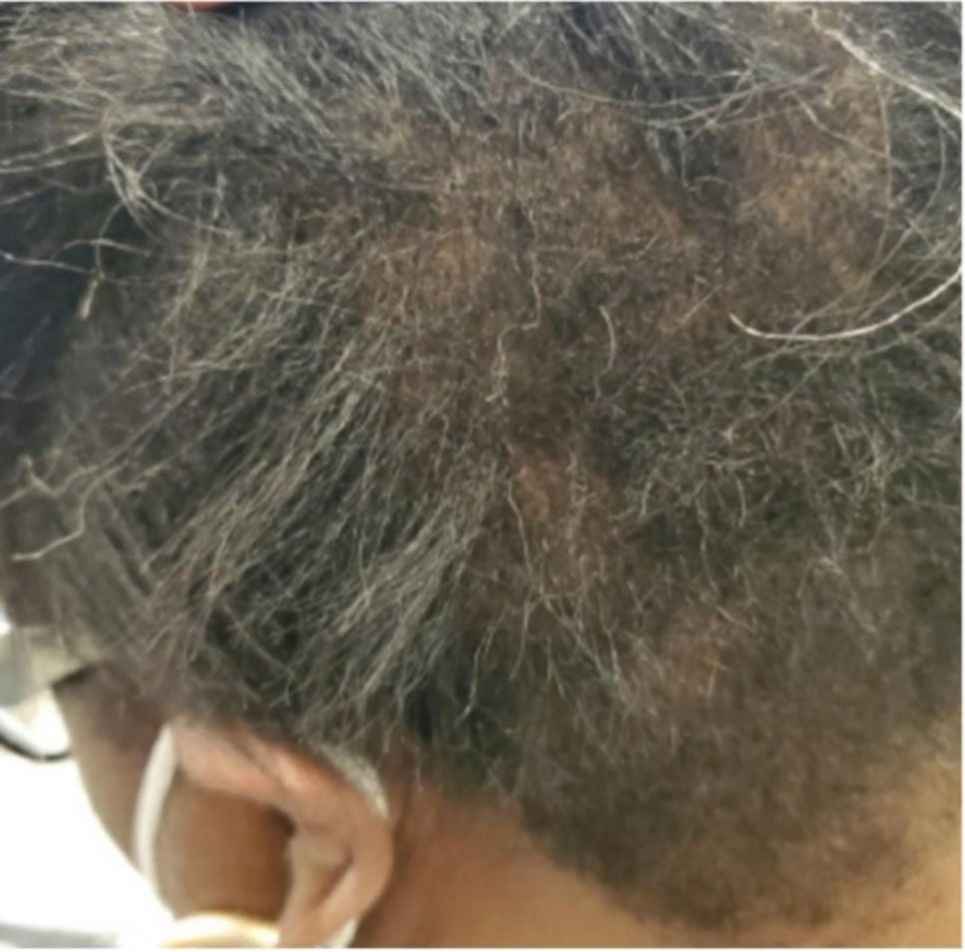
Occipital hair breakage

Patients present with short, broken hairs of normal diameter (Fig. 11). Seborrheic dermatitis associated with scalp pruritis can present as hair breakage caused by scratching and ultimate breakage of hair shafts [23]. A hair pull test or tug test will be positive for short hair fragments that lack roots or breakage of hair shafts at variable distances from the scalp [19].

##### Trichoscopy and pathology

Trichoscopy findings include areas of swelling or nodular thickenings along the hair shaft, indicating weakening at the nodes, and uneven breakages resembling broomstick-like projections [22, 26], that appear lighter in color compared with the remaining hair shafts [26]. Biopsy is rarely indicated if the underlying scalp is normal. If there is lichenification or evidence of follicular dropout on clinical/trichoscopic exam, a biopsy to rule out underlying traction or scarring alopecia is indicated. Patients can present with bubble hair, characterized by air-filled spaces in the hair shaft, associated with overheating the hair shaft with blow dryers, flat or curling irons. This can be visualized with electron microscopy [27].

##### Differential diagnosis

Hair breakage or weathering along the occipital scalp can be misdiagnosed as TC, therefore a confirmation with a KOH test should be performed [24].

### Scarring conditions

#### Lichen planopilaris

LPP, a follicular variant of lichen planus (LP), is an inflammatory, lymphatic disease affecting the scalp, primarily the vertex and parietal areas [28]. This disorder classically produces asymmetric multifocal areas of scarring alopecia that coalesce into larger areas of hair loss, perifollicular erythema and keratotic follicular papules [29]. Although, the etiology is not clear, it is thought to involve an autoimmune process, in which the hair follicles are targeted predominantly by lymphocytes and neutrophils leading to destruction of hair follicle stem cells [28].

##### Clinical exam

Patients present with perifollicular erythema and hyperkeratosis. Symptoms, if present, range from mild to severe, with pruritus, burning, or trichodynia [12]. Cutaneous, nail, and mucous membrane LP can occur at any point in the disease process [29]. Positive anagen pull test indicates active disease [28].

##### Trichoscopy and pathology

On trichoscopy, active lesions may have intense perifollicular hyperkeratosis, diffuse milky-red areas, targetoid bluish-gray pigmentation, perifollicular casts, or loss of follicular ostia, peripilar white scales, and peripilar erythema [28]. Inactive disease shows small, irregularly shaped, whitish areas lacking follicular openings and hypopigmented areas of fibrosis [28]. Histopathological findings reveal perifollicular lymphohistiocytic infiltrates, sometimes with a lichenoid pattern, more prominent in the isthmus and infundibulum regions, vacuolar degeneration of basal cells, necrotic keratinocytes, and artifactual clefts between the follicle and the perifollicular fibrous band; perifollicular fibrosis can be seen separating the inflammatory infiltrate from the follicle [30]. There is erythema around the follicle at the periphery and as hair follicles are replaced by fibrous tissue, the inflammation ceases [28]. Over time, there is a reduction and loss of sebaceous glands and destruction of the entire hair follicle, which is replaced by fibrous tissue [28].

##### Differential diagnosis

Diagnosing LPP can be difficult as it is often mistaken for seborrheic dermatitis, discoid lupus erythematosus, and CCCA.

#### Frontal fibrosing alopecia

FFA, a primary lymphocytic scarring alopecia, is a well-characterized variant of LPP [8, 12]. While the etiology remains unknown, genetic, hormonal, and autoimmune factors likely play a role, leading to follicle stem cell destruction and loss of regenerative potential of the hair follicle, resulting in fibrosis [31]. FFA is common in Caucasian postmenopausal women, though cases have been reported in all ethnicities and adult female age groups [29, 31]. It is characterized by a progressively receding frontotemporal and parietal hairline with scarring that often includes eyebrow loss [31]. Though not commonly recognized, the occipital scalp may also be affected, which is known as ophiasis pattern FFA [32].

##### Clinical exam

**Fig. 12:**
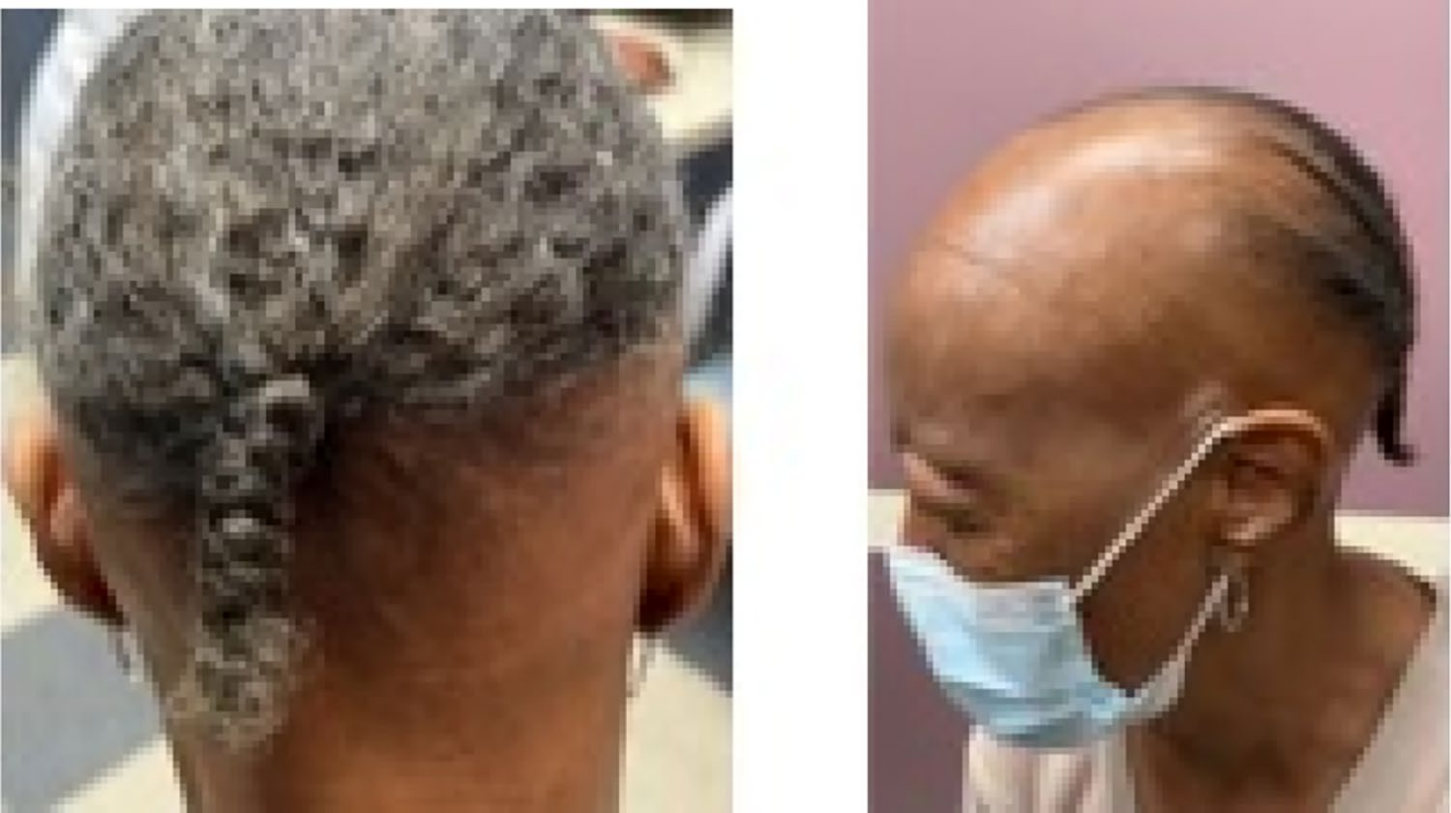
Occipital frontal fibrosing alopecia

**Fig. 13:**
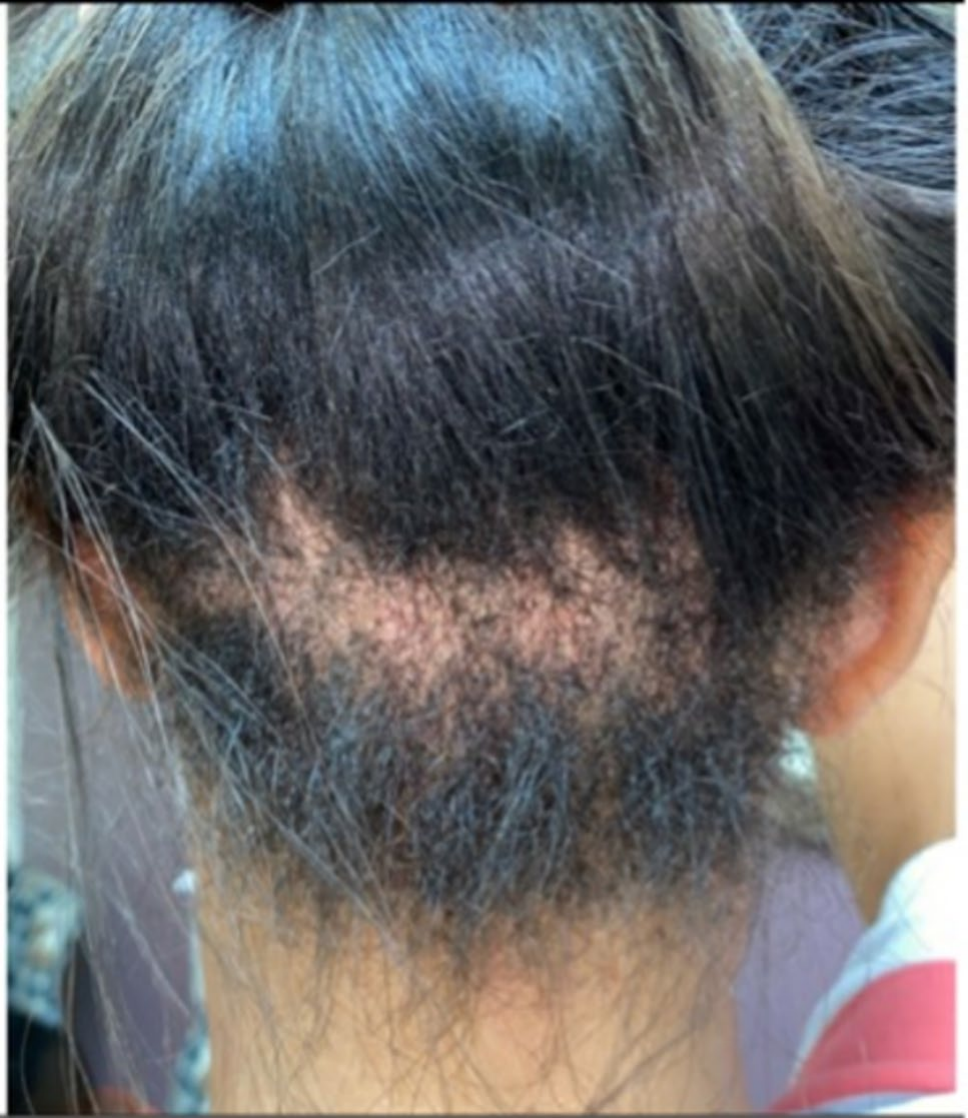
Occipital frontal fibrosing alopecia

Patients present with pruritus, pain and burning, though less common in occipital scalp involvement [32]. Patients present with a progressive band of hair loss affecting the frontal and/or temporal hairline, often accompanied by eyebrow alopecia [31], or yellowish facial papules and pigmented skin patches [33] (Figs. 12, 13). The lonely hair sign has been described, but a pseudo-fringe may be seen at the frontal hairline [34], along with a positive hair pull test. Depression of the frontal veins, presence of increased preauricular lines, follicular re-pigmentation of the white/grey hair in the frontal, temporal, and occipital hairline can be seen [33].

##### Trichoscopy and pathology

**Fig. 14:**
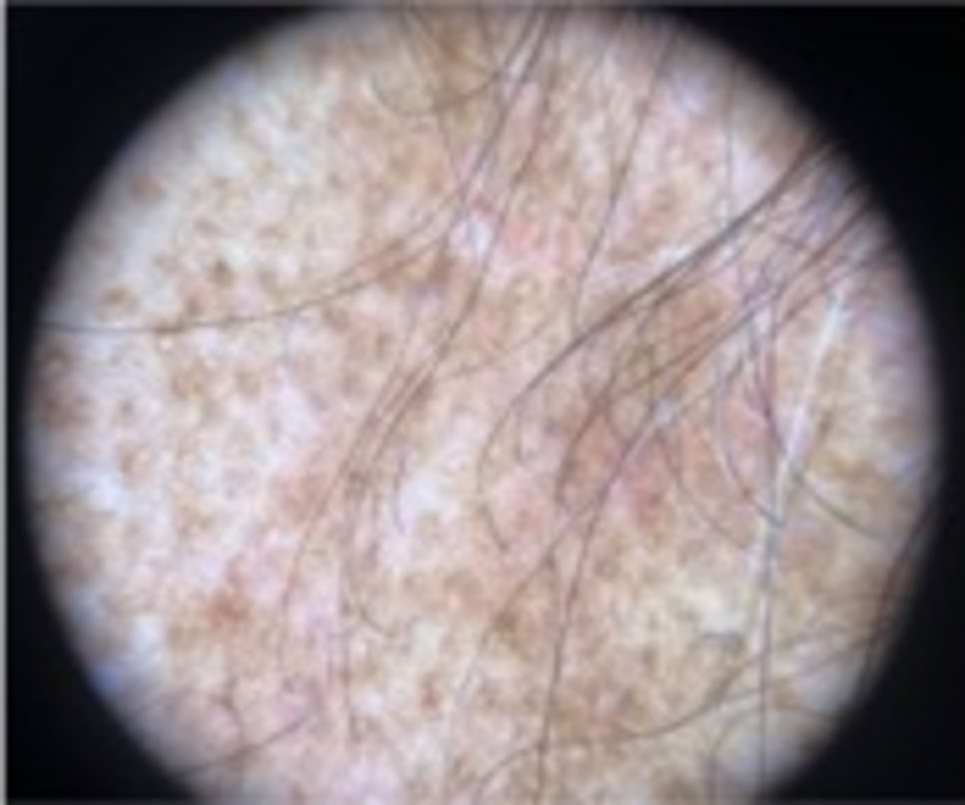
Perifollicular erythema, mild perifollicular scale, absence of follicular openings, hypopigmented cicatricial zones, lonely hairs, ivory-beige background in frontal fibrosing alopecia

**Fig. 15:**
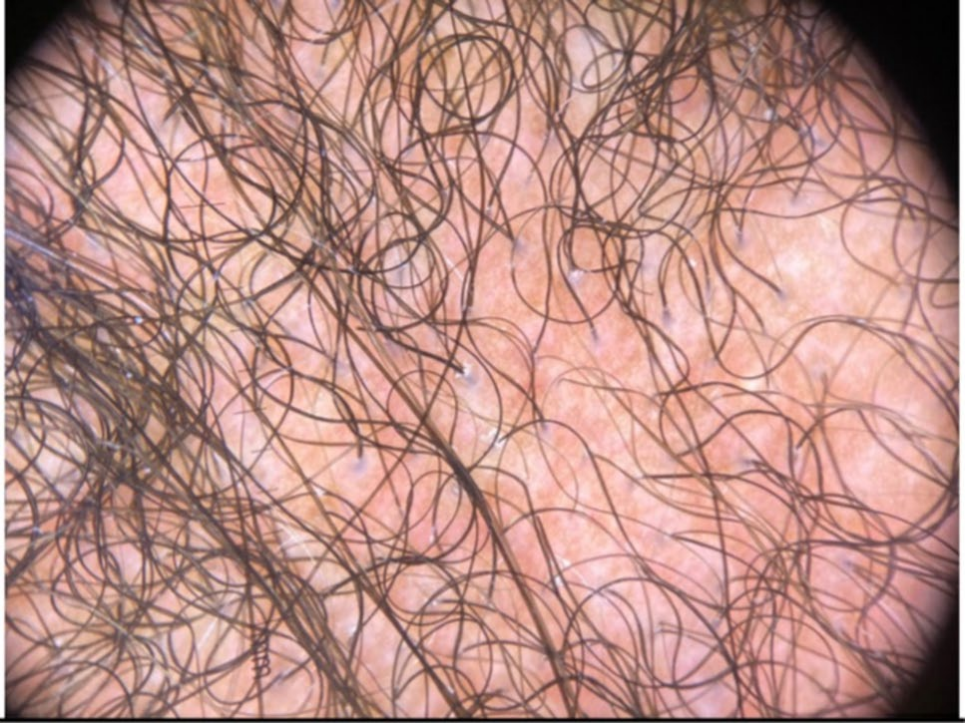
Perifollicular erythema, mild perifollicular scale, absence of follicular openings, hypopigmented cicatricial zones in frontal fibrosing alopecia

Trichoscopy findings include perifollicular erythema, mild perifollicular scale, absence of follicular openings, hypopigmented cicatricial zones, lonely hairs, ivory-white or ivory-beige background, absence of vellus hairs in the frontal hairline, peripilar casts, and single-terminal hairs at the hair bearing margin [34] (Figs. 14, 15). Early stages show mild perifollicular fibrosis, perifollicular lymphohistiocytic lichenoid inflammation in the infundibulum, isthmus, and bulge area regions with spared interfollicular epidermis [30, 31]. Late stages have severe perifollicular fibrosis, follicular dropout, absence of sebaceous glands, and apoptotic cells in the outer root sheath, with reduced follicular density until scar tissue replaces the pilosebaceous units [29]. Lastly, the external outer root sheath shows vacuolar degeneration of the basal layer, mild keratinocyte degeneration, eosinophilic necrosis of cells, and sparing of the interfollicular epidermis [30, 31]. Specifically, occipital scalp findings include diffuse erythema, peripilar erythema, peripilar scaling, black dots, pili torti, circular hairs, and no yellow dots [32].

##### Differential diagnosis

FFA is commonly mistaken as TA in Black patients [35]. Clues to aid in prompt diagnosis include early onset, facial hyperpigmentation, presumed to be lichen planus pigmentosus, and pruritus, which indicates a greater inflammatory response [35]. Additionally, scale is less prominent and hairline recession is more common in Black patients [35] with more vertex/central and occipital scalp involvement compared to white patients [35]. The presence of follicular dropout helps differentiate FFA from TA and AA, which is absent in FFA, decreased in TA, and retained in AA. The absence of vellus hairs and sebaceous glands distinguishes FFA from TA.

#### Central centrifugal cicatricial alopecia

CCCA, commonly seen in women of African descent, is a chronic progressive primary lymphocytic scarring alopecia [36, 37]. Etiology is unknown, however, contributing factors include genetics, infections, methods of hair grooming, association with female pattern alopecia, and mechanical factors, such as heat and traction [36, 37]. Classically, hair loss begins on the crown and spreads peripherally, typically sparing the lateral and posterior scalp [8, 36]. Recent reports demonstrate CCCA affecting the lateral and posterior aspects of the scalp [38, 39]. One study of women with biopsy and trichoscopic proven vertex CCCA showed dermatoscopic and histopathological evidence of CCCA seen far beyond the vertex of the scalp, despite little to no clinical evidence of hair loss in those areas [40].

##### Clinical exam

**Fig. 16:**
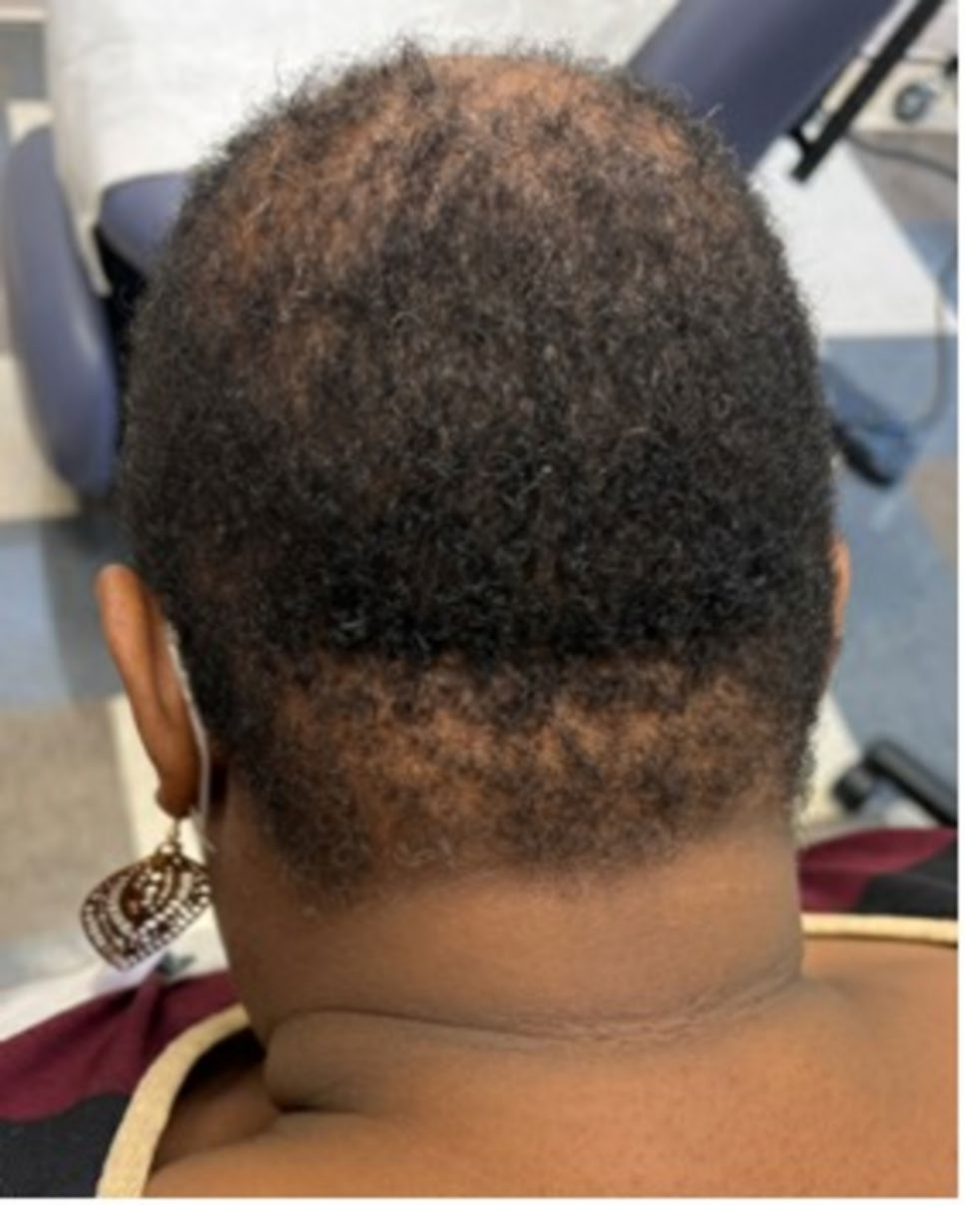
Occipital central centrifugal cicatricial alopecia

A first early sign is hair breakage or thinning with mild symptoms of pruritis, pain, or tenderness [37, 38], or no symptoms at all [36]. Patients present with chronic, progressive central scalp hair loss that expands centrifugally symmetrically [36] (Fig. 16). Advanced cases show a smooth and shiny scalp with follicular dropout [36].

##### Trichoscopy and pathology

Trichoscopy shows peripilar white–gray halos, perifollicular hyperkeratosis, and follicular dropout. Early histopathological findings include perifollicular lymphocytic infiltrate and perifollicular fibroplasia involving the infundibulum and isthmus. There is also a reduction in terminal hair follicles with an increase in fibrous tracts and premature desquamation of the inner root sheath [12, 36, 38]. With progression and no intervention, late histological findings show loss of sebaceous glands, perifollicular fibrosis, dermal scarring, and dermal lymphocytic and plasma cell infiltrate [12, 36].

##### Differential diagnosis

Differential diagnosis includes LPP, FFA, FD, DLE, female and male pattern alopecia, TA, and TC. Some of these are distinguishable using trichoscopy or histopathology.

#### Acne keloidalis nuchae

AKN, a chronic inflammatory skin condition, is characterized by the development of keloid like papules, pustules, and plaques on the occipital scalp and posterior neck [41]. Etiology is unknown, however, contributing factors include chronic irritation or occlusion of the follicles due to hair cutting practices, trauma, friction, heat, humidity, and infection [41] AKN commonly begins after puberty with persistent folliculitis at the nape of the neck that can ultimately lead to cicatricial alopecia [41]. Onset is generally preceded by pruritus, pain, bleeding a few hours to days after a haircut, or mild irritation from use of a headband or helmet [41].

##### Clinical exam

**Fig. 17:**
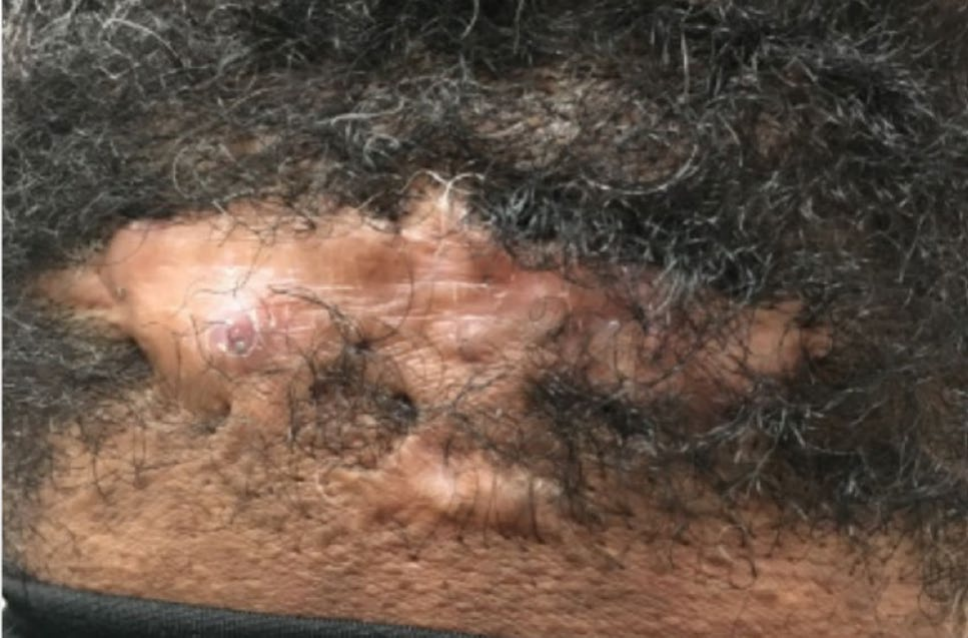
Acne keloidalis nuchae

Patients present with firm, dome-shaped, inflammatory papules and pustules over the nape of the neck [42]. (Fig. 17) Early stages include inflamed, irritated small papules and notable erythema; papules may become secondarily infected and develop into pustules and or abscesses [41]. Late stages show widespread fibrosis and scar formation as papules coalesce into larger plaques, nodules or a tumor-like masses often encompassing the entire back of head, extending to the vertex scalp and neck [42]. Variable sized keloidal papules or nodules at the site of previously involved hair follicles may be present [42].

##### Trichoscopy and pathology

Trichoscopy findings include hair tufts trapped in individual papules coalescing together forming larger lesions [41]. There is loss of follicular ostia, absent ingrown hairs, perifollicular pustules, and white halos surrounding hair follicles corresponding to perifollicular fibrosis [41]. Biopsy is rarely needed, but findings include neutrophils and lymphocytes distributed around the isthmus and lower infundibulum, lamellar fibroplasia, and naked hair shafts in the dermis [12, 42]. Thinning of the follicular epithelium is seen at the isthmus and several hairs are seen sharing the same infundibulum [41]. As this progresses, there is destruction of the sebaceous glands and chronic granulomatous inflammation with evidence of collagen deposition and fibrosis [12, 42].

##### Differential diagnosis

AKN can be misdiagnosed as bacterial folliculitis, acne vulgaris or conglobata, FD, DC, or hidradenitis suppurativa (HS). The absence of comedones can rule out acne.

#### Dissecting cellulitis

DC, a rare, chronic, and progressive disease, is primarily seen in middle-aged males of African descent. DC affects the vertex and occiput, beginning as folliculitis and expanding into abscesses with purulent drainage and sinus tract formation. DC is included in the “follicular occlusion tetrad,” along with pilonidal cysts, HS, and acne conglobata, suggesting a common pathogenesis involving deep follicular occlusion and infection [43, 44].

##### Clinical exam

**Fig. 18:**
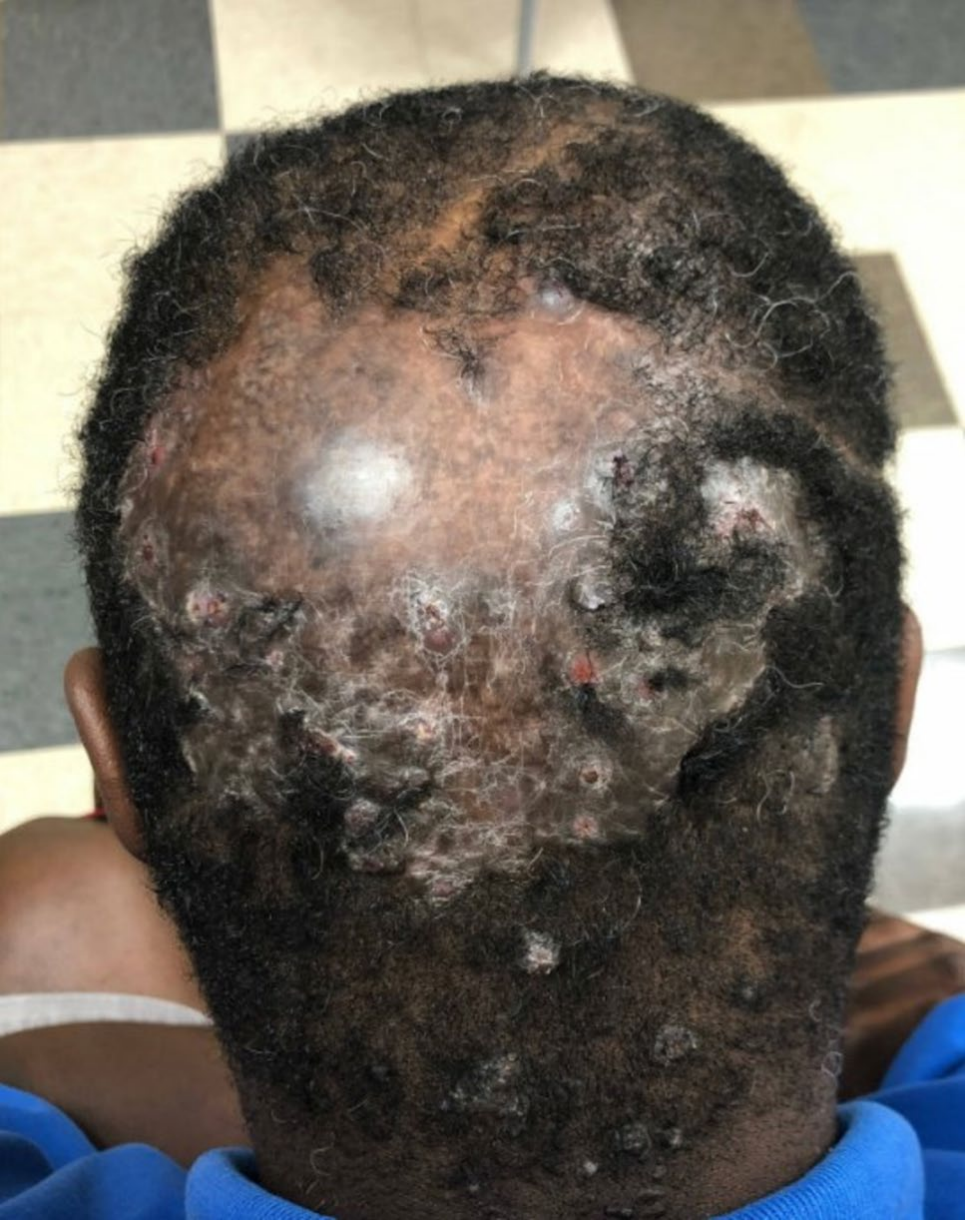
Dissecting cellulitis

Clinical findings vary with extent and severity of the disease. In 2018, a severity-based classification was proposed for DC consisting of three different clinical-pathological stages [44]. Stage 1 is characterized by isolated nodules/abscesses, separated by normal skin, without intercommunicating sinus tract or scarring alopecia [44]. Stage 2, the abscedens stage, is characterized by nodules/abscesses with intercommunicating sinus tract without scarring [44]. Stage 3, the fibrotic stage, is characterized by nodules or abscesses with scarring or permanent alopecia [44] (Fig. 18).

##### Trichoscopy and pathology

Trichoscopic findings in stage 1 may simulate that of AA with the presence of follicular and perifollicular lymphocytic infiltrates on the lower parts of terminal follicles, yellow dots, broken hairs, black dots, and exclamation mark hairs [45]. Stage 2 includes three-dimensional yellow dots with “soap bubble”-like appearance, yellow structureless areas known as “lakes of pus,” and pinpoint-like vessels with a whitish halo [45]. Stage 3 shows white areas lacking follicular openings that represent tissue fibrosis [45]. Additionally, cutaneous clefts with emerging hairs may develop [45]. Biopsy findings include a predominance of neutrophilic infiltrate. Later stages reveal extensive dermal fibrosis and destruction of sebaceous glands [45].

##### Differential diagnosis

DC can be misdiagnosed as AKN, FD, TC, cellulitis, and pilar cysts.

#### Folliculitis decalvans

FD, a rare inflammatory presentation of cicatrizing alopecia, primarily seen in young and middle-aged Black men. It is characterized by inflammatory perifollicular papules and pustules affecting the occipital and vertex scalp. Etiology is unknown, however, contributing factors include *Staphylococcal aureus* and genetics [46, 47].

##### Clinical exam

**Fig. 19:**
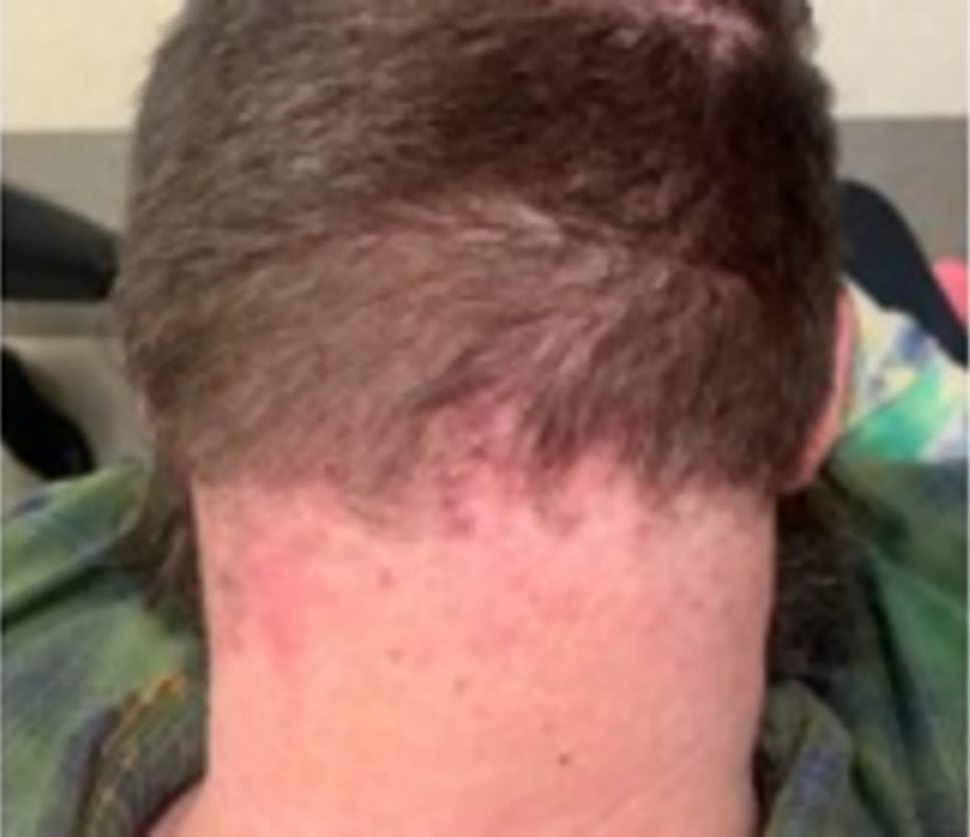
Folliculitis decalvans

Clinical findings include the development of scarred areas and areas of follicular pustules [46, 47]. (Fig. 19) Additionally, erythema and yellow-gray scales around follicles, follicular keratosis, erosions, and hemorrhagic crusts can be seen [46, 47]. Patients can present with spontaneous bleeding, pain, itching or a burning sensation. As the disease progresses, irregularly shaped, atrophic flesh-colored or ivory-white patches of cicatricial alopecia develop, which tend to be thicker and more indurated compared to other scarring scalp disorders [46]. A common finding is tufted folliculitis, characterized by 5–20 hairs emerging from one single dilated follicular orifice, known as polytrichia [46, 47].

##### Trichoscopy and pathology

**Fig. 20:**
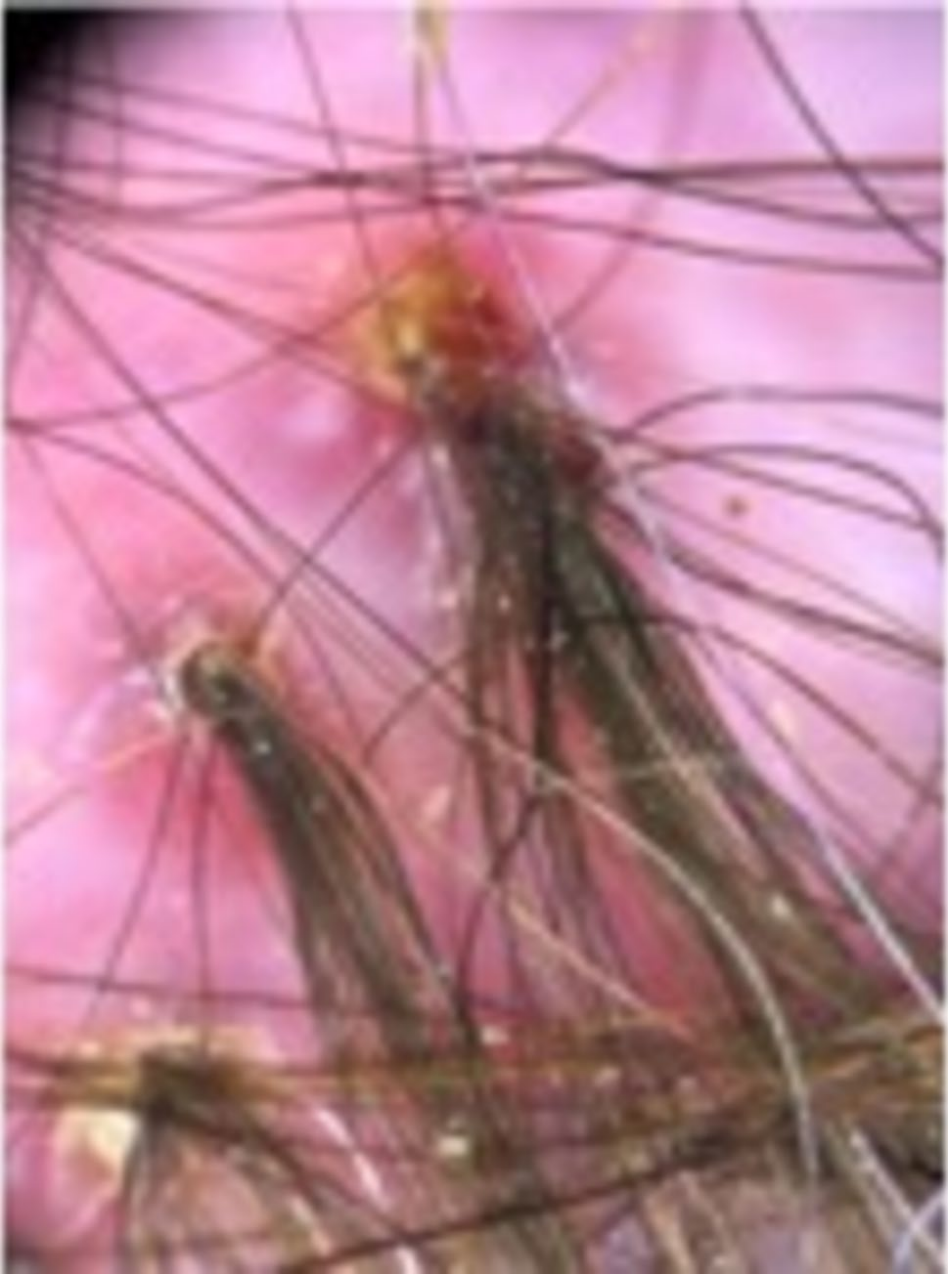
Follicular tufts, perifollicular erythema in a starburst pattern, yellowish tubular scaling, crusts, and pustules. Milky-red areas without follicular orifices in folliculitis decalvans

Tricoscopic findings include follicular tufts, perifollicular erythema in a starburst pattern, yellowish tubular scaling, crusts, and pustules [47]. Late stages show ivory-white and milky-red areas without follicular orifices [47] (Fig. 20). Histopathologically, FD is characterized as a neutrophilic primary cicatricial alopecia. Early stages show keratin aggregation and a dilatation of the infundibulum combined with numerous intraluminal neutrophils, the destruction of sebaceous glands, and a predominant neutrophilic, intrafollicular and perifollicular infiltrate [46, 47]. In later stages, this infiltrate can consist of neutrophils, lymphocytes and numerous plasma cells extending into the dermis, and around the blood vessels of the superficial and mid-dermis [46, 47]. Other findings include granulomatous inflammation with foreign-body giant cells, fibrous tracts in the area of the former follicle, interstitial dermal fibrosis, and/or hypertrophic scarring [46].

##### Differential diagnosis

FD can be misdiagnosed as TC, DC, acne necrotica, folliculitis, and erosive pustular dermatosis. The absence of sinus tracts can distinguish FD from DC.

## Conclusion

There are multiple forms of hair loss, both scarring and non-scarring, that affect different parts of the scalp. This review primarily focuses on conditions that can affect the posterior scalp. The etiology of hair loss affecting this area is unknown, however, we can theorize some explanations that include irritation of the hair follicles due to movement, frequent occlusion of the area, and a possible predilection that these hair follicles are at risk in a way that we do not understand. In this review, we described forms of posterior hair loss and provided a differential given that these conditions may be difficult to diagnose due to their presentation in this area. As a result, these conditions are often misdiagnosed or delayed in diagnosing, which can lead to poor outcomes and a negative effect on quality of life. In this review, we highlight the clinical findings, trichoscopic findings, and histopathological findings of different forms of hair loss that often occur solely on the occiput, for physicians to interpret and make an accurate diagnosis. We also highlight some distinguishing factors of occipital scalp conditions for comparison (Table 1). We hope that this review will aid in diagnosing these conditions promptly so that patients can begin the most appropriate treatment plan with the best prognosis. Additionally, it is common for these conditions to co-exist together, so the identification of each condition is paramount to the care of patients.

## Data Availability

No datasets were generated or analysed during the current study.
